# Association between Dietary Macronutrient Intake and Symptoms in Uninvestigated Dyspepsia: Evidence from a Population-Based, Cross-Sectional Study

**DOI:** 10.3390/nu14132577

**Published:** 2022-06-22

**Authors:** Shahram Agah, Azadeh Aminianfar, Ammar Hassanzadeh Keshteli, Vida Bitarafan, Peyman Adibi, Ahmad Esmaillzadeh, Christine Feinle-Bisset

**Affiliations:** 1 Colorectal Research Centre, Iran University of Medical Sciences, Tehran 1445613131, Iran; shahramagah@gmail.com; 2 Adelaide Medical School, University of Adelaide, Adelaide, SA 5005, Australia; vida.bitarafan@adelaide.edu.au; 3 Research Centre for Biochemistry and Nutrition in Metabolic Diseases, Kashan University of Medical Sciences, Kashan 8715988141, Iran; aminian127@gmail.com; 4 Department of Medicine, University of Alberta, Edmonton, AB T6G 2P5, Canada; akeshteli@gmail.com; 5 Integrative Functional Gastroenterology Research Centre, Isfahan University of Medical Sciences, Isfahan 8174673461, Iran; 6 Centre of Research Excellence in Translating Nutritional Sciences to Good Health, University of Adelaide, Adelaide, SA 5005, Australia; 7 Gastroenterology and Hepatology Research Centre, Isfahan University of Medical Sciences, Isfahan 8174673461, Iran; payman.adibi@gmail.com; 8 Department of Community Nutrition, School of Nutritional Sciences and Dietetics, Tehran University of Medical Sciences, Tehran 1416634793, Iran; 9 Obesity and Eating Habits Research Centre, Endocrinology and Metabolism Molecular-Cellular Sciences Institute, Tehran University of Medical Sciences, Tehran 1416634793, Iran; 10 Department of Community Nutrition, Isfahan University of Medical Sciences, Isfahan 8174673461, Iran

**Keywords:** diet, macronutrient, fat, carbohydrate, protein, uninvestigated dyspepsia, dyspeptic symptoms, postprandial fullness, epigastric pain, early satiation

## Abstract

(1) Background: Limited evidence from laboratory-based studies suggests that specific dietary macronutrients, particularly fat, can induce dyspeptic symptoms. Through a population-based study, we investigated the relationship between dietary macronutrients and dyspeptic symptoms and sought to determine macronutrient intake thresholds to predict or prevent dyspepsia and reduce symptoms in patients with dyspepsia. (2) Methods: A total of 4763 Iranian people were enrolled in this population-based, cross-sectional study. Uninvestigated dyspepsia (UD) and its symptoms, including postprandial fullness, early satiation, and epigastric pain, were evaluated using a modified Persian version of the Rome III criteria. The dietary intakes of participants were evaluated using a validated food–frequency questionnaire. Receiver operating characteristic (ROC) curve analysis was used to calculate threshold intakes of dietary macronutrients to prevent UD in the general population. The analysis was then repeated in those with UD to calculate intake thresholds for reducing UD symptoms. (3) Results: Early satiation occurred in 6.3% (n = 302), postprandial fullness in 8.0% (n = 384) and epigastric pain in 7.8% (n = 371) of participants. The prevalence of UD was 15.2%. Compared with individuals without UD, those with UD had a lower intake of carbohydrates (48.2% vs. 49.1%) and a higher intake of fats (38.3% vs. 37.4%), while protein and energy intakes did not differ. Higher dietary fat and protein intakes were associated with a higher prevalence of postprandial fullness and epigastric pain, respectively. Macronutrient intakes to *predict* UD in the general population were <49% of energy from carbohydrates, >14.7% from protein, and >37.7% from fats. Carbohydrate, protein, and fat intakes to *prevent* symptoms among those with UD were calculated to be >48.2%, <14.6%, and <38.6%, respectively. (4) Conclusion: Higher carbohydrate intake and lower fat or protein intakes were associated with a lower likelihood of UD. Prospective studies carefully manipulating dietary macronutrient composition are warranted to investigate the value of dietary changes to improve symptoms in people with UD.

## 1. Introduction

Functional dyspepsia (FD) is one of the most common functional gastrointestinal (GI) disorders (now termed ‘disorders of gut–brain interaction’), characterised by chronic and recurrent symptoms, predominantly in the upper GI tract, in both developed and developing countries [1,2,3]. It has a major negative impact on the physical, social, and mental health of patients and represents a substantial economic burden on the healthcare system [4,5,6]. The prevalence of FD has been reported to be ~20% in studies conducted on different study populations across the world [2,7]. In this context, it is important to recognise that, in most previous epidemiological studies, the disorder was not diagnosed after appropriate clinical investigation but based on the GI symptoms reported by individuals; hence, it would be more appropriate to classify the disorder in this context as ‘uninvestigated’ dyspepsia (UD). However, over three-quarters of individuals who undergo clinical and endoscopic evaluation are then diagnosed with FD, based on a lack of abnormal findings in endoscopy (including peptic ulcer, erosive gastroduodenitis, etc.) [8], while ~5% of individuals with UD were found to have a gastric ulcer, and ~0.5% may be diagnosed with gastric cancer, following endoscopic evaluation, based on a recent study from the Middle-East [9]. Thus, it appears that the majority of people with UD may be regarded as having FD.

Although the pathophysiology of dyspepsia is multifactorial, many patients report that their symptoms occur, or are exacerbated, after food ingestion. Several studies have shown a possible role of dietary factors (e.g., caloric intake, eating patterns, specific foods or food components, or macronutrient composition) in the development of dyspepsia [10,11]. Fatty foods, in particular, are frequently identified as culprits by patients. Supporting this finding, a recent systematic review of 16 clinical trials and observational studies reported that dietary fat may play a key role in the development of FD-related symptoms [12].

To date, most clinical studies evaluating the effects of diet or specific dietary nutrients on symptoms in FD have been performed in relatively small sample sizes [13,14,15,16,17,18], making the characterisation of relationships or identification of subtle effects difficult. Thus, larger trials including large sample sizes across a diverse range of participants would (1) be more likely to identify more modest relationships between macronutrient consumption and symptom occurrence and (2) potentially allow the calculation of limits of consumption of a particular nutrient to prevent symptoms, of which small trials are not capable. Therefore, the aim of the present study was to assess the association(s) between dietary macronutrient intake with dyspeptic symptoms in a large sample of Iranian adults. Furthermore, we aimed to determine the macronutrient composition of a diet that may predict or prevent the occurrence of dyspeptic symptoms.

## 2. Materials and Methods

### 2.1. Study Population

The data analysed in this study were obtained from the Study on the Epidemiology of Psychological, Alimentary Health and Nutrition (SEPAHAN) project, which included Iranian adults working in 50 different health centres in Isfahan Province, Iran. Detailed information about the design, study participants, and data collection has been published previously [19]. Briefly, in the first phase of SEPAHAN, demographic, lifestyle, and nutritional data were collected using a self-administered questionnaire which was sent to individuals aged 18 to 55 years; of 10,087 individuals contacted, 8691 returned the completed questionnaires (response rate: 86.2%). We excluded individuals with implausible daily energy intakes (>4200 or <800 kcal/d) to minimise influences due to under- or over-reporting (n = 500). We also excluded individuals with missing data. In the second phase, participants were invited to complete questionnaires relating to GI function and their psychological status; among the 9652 subjects invited, 6239 completed the questionnaires (response rate: 64.6%). For the purpose of the current analysis, we used the data from 4763 adults who had provided complete information about both dietary intake and habits and GI function in the two phases of the study. The study protocol was approved by the Bioethics Committee at Isfahan University of Medical Sciences [19]. Moreover, written informed consent was obtained from each participant before their inclusion.

### 2.2. Assessment of Dyspepsia

The presence of dyspeptic symptoms was determined using a modified Persian version of the Rome III questionnaire [19]. The validity and reliability of this questionnaire in an Iranian population were established previously [20]. Accordingly, we defined UD as the presence of one or more upper GI symptoms, including bothersome postprandial fullness (a feeling of uncomfortable fullness after a regular-sized meal), early satiation (an inability to finish a normal-sized meal), and/or epigastric pain or epigastric burning (feeling pain or burning in the upper abdomen), often or always, during the last three months. The severity of symptoms was not evaluated. Information as to whether individuals had sought healthcare advice for their symptoms was not obtained.

### 2.3. Assessment of Dietary Intake

Dietary intake was assessed using an adjusted 106-item, dish-based, semi-quantitative food–frequency questionnaire (FFQ) [21,22]. Briefly, this questionnaire assessed five main components—namely, main dishes; grain-based foods and potatoes; dairy products; fruit and vegetables; and miscellaneous food items (e.g., sweets, fast foods, nuts, desserts) and beverages. Portion sizes were used for the determination of the amount of food consumed, which was subsequently converted to grams per day using household measures [23]. Daily nutrient intakes of participants were estimated based on the United States Department of Agriculture food consumption database [24]. The validity and reliability of the FFQ were examined previously in a subgroup of 200 randomly selected participants of the SEPAHAN project. For this purpose, the newly designed FFQ was completed at baseline and six months later. During the six-month period, participants provided three detailed dietary records that were used as the gold standard. Based on those findings, the FFQ provides a reasonably reliable measure of long-term dietary intake in the Iranian population; for instance, dietary carbohydrate intake, as obtained from the FFQ, was highly correlated with the intake, as obtained from the average of three dietary records (r = 0.81) [22].

### 2.4. Assessment of Other Variables

Self-administered questionnaires were used to collect information on age, sex, marital status (single/married), education (high school diploma or below/above high school diploma), smoking habits (non-smoker/former smoker/current smoker), family size (≤4/>4 members) and homeownership (owner/non-owner). Socioeconomic status (SES) was calculated using family size, education status, and homeownership. Participants were given a score of 1 if they had a family size of ≤4, academic education, and homeownership. If their family size was >4, or they had non-academic education or were not homeowners, they were given a score of 0. Then, these scores were added, to obtain an SES score of 0 (poor), 1 (middle class), or 2 or more (high). Data on anthropometric measures (height, weight, and waist circumference) were collected using a self-reported questionnaire. The body mass index (BMI) of each study participant was calculated as weight in kilograms divided by height in metres squared and then categorised into three classes: normal weight, overweight, and obese. The physical activity of study participants was assessed using the General Practice Physical Activity Questionnaire (GPPAQ) [25]. This questionnaire provides a simple, four-level physical activity index (PAI) to rank each individual’s current physical activity, and we categorised participants into four groups: active (>3 h/week), moderately active (1–3 h/week), moderately inactive (<1 h/week), and inactive (no physical activity) [25].

### 2.5. Statistical Analysis

General characteristics of participants with or without UD are expressed as means ± SDs for continuous variables and as percentages for categorical variables. Participants were categorised based on current recommended levels of macronutrient intake as follows: carbohydrates: ≤50%, 50–55%, and >55% of energy intake; proteins: ≤15%, 15–20%, and >20% of energy intake; and fats: ≤25%, 25–35%, and >35% of energy intake. To examine the differences between participants with or without UD, we used an independent Student’s *t*-test for continuous variables and a chi-square test for categorical variables. The prevalence of UD and its symptoms across categories of macronutrient intakes were tested using the chi-square test. Binary logistic regression was used to estimate ORs and 95% CIs for the presence of UD, postprandial fullness, early satiation, and epigastric pain across categories of macronutrient intakes in crude and multivariable-adjusted models, in which we adjusted for age, sex, energy intake, physical activity, marriage status, education, socioeconomic status, smoking, and BMI. P for trends was determined by considering categories of macronutrient intakes as linear continuous variables in the logistic regression analysis. Receiver operating characteristic (ROC) curve analysis was performed to calculate the area under curves (AUCs) of macronutrient intakes to *predict* UD and its symptoms in the general population. The ROC curve is a plot to identify the diagnostic ability of a continuous variable for a binary outcome variable. The curve assesses the sensitivity and specificity of each point in the continuous variable to detect the outcome, and the point of maximum sensitivity and specificity in the continuous variable predicts the outcome variable. To calculate these points or cutoff values, we considered each individual macronutrient (every % intake of each carbohydrate, fat, or protein) separately as the independent continuous variable in the ROC curve analysis and UD as the binary outcome variable. The sensitivity and specificity of each % energy for each macronutrient were then computed, and the point of maximum sensitivity and specificity was selected as the appropriate cutoff to predict UD. In an attempt to calculate macronutrient intakes for the *prevention* of UD and its symptoms, the above analyses were repeated but restricted to people with UD. All statistical analyses were performed using the Statistical Package for Social Sciences (version 20; SPSS Inc., Chicago, IL, USA). *p* < 0.05 was considered statistically significant.

## 3. Results

The prevalence of UD was 15.2% in the study population. Early satiation occurred in 6.3% (n = 302), bothersome postprandial fullness in 8.0% (n = 384), and epigastric pain in 7.8% (n = 371). General characteristics of the study population, by the status of UD, are provided in Table 1. Participants with UD were more likely to be female, less likely to be university graduates or have a high socioeconomic status, and had lower mean weight and BMI than those without UD. No significant differences were found in terms of age, marital status, smoking, or physical activity between participants with or without UD.

### 3.1. Dietary Intakes

Dietary intakes, by the status of UD, are shown in Table 2. Participants with UD had a lower dietary intake of carbohydrates (*p* = 0.01) and a higher intake of fats (*p* = 0.007). No significant differences were found for intakes of protein or energy.

### 3.2. Prevalence of UD and Symptoms and Their Correlations with Macronutrient Intakes

No significant association was found between the % energy of intake of any macronutrient and the prevalence of UD (Table 3). There was, however, a significant association between postprandial fullness and the intake of fat. Individuals with the highest dietary fat intake were more likely to have postprandial fullness, compared with those with the lowest intake (38.6% vs. 28.3%, *p* = 0.04). In addition, epigastric pain was more prevalent in participants with the greatest intake of proteins than in those with the lowest intake (50% vs. 39.7%, *p* = 0.02). No other associations were found.

Multivariable-adjusted ORs and 95% CIs for UD and its symptoms across categories of macronutrient intakes are shown in Table 4. Individuals who consumed >55% of energy from carbohydrates had a non-significantly decreased risk of UD than those who consumed ≤50%, either before (OR: 0.84; 95% CIs: 0.66, 1.08, *p*-trend = 0.15) or after (0.86; 95% CIs: 0.64, 1.16, *p*-trend = 0.37), controlling for potential confounders. Individuals who consumed >20% of energy from protein had a non-significantly increased odds of UD than those who consumed ≤15%, either before (OR: 1.63; 95% CIs: 0.95, 2.70, *p*-trend = 0.81) or after (1.35; 95% CIs: 0.65, 2.77, *p*-trend = 0.61), controlling for potential confounders. Moreover, after controlling for potential confounders, people with a fat intake of >35% had a non-significantly increased risk of UD than those with a fat intake of ≤25% (OR: 1.19; 95% CIs: 0.58, 2.46, *p*-trend = 0.30).

### 3.3. Area under ROC Curve Values for Macronutrients to Predict UD

Areas under the ROC curve (AUCs) for different macronutrients to *predict* UD and symptoms are shown in Table 5. AUC for carbohydrate intake to *predict* UD was 0.47 (95% CI: 0.44, 0.49), and AUCs for postprandial fullness, early satiation, or epigastric pain were 0.48, 0.47, and 0.49, respectively. AUC for protein intake to *predict* UD was 0.48 (95% CI: 0.48, 0.51), and AUCs for postprandial fullness, early satiation, or epigastric pain were 0.48, 0.48, and 0.47, respectively. AUC for dietary fat intake to *predict* UD was 0.53 (95% CI: 0.50, 0.56), and AUCs for postprandial fullness, early satiation, and epigastric pain were 0.52, 0.53, and 0.50, respectively.

### 3.4. Calculated Levels of Macronutrients to Predict UD and Its Symptoms

Calculated macronutrient intakes to *predict* UD and its symptoms in the whole population are provided in Table 6. Thus, macronutrient intakes to *predict* UD, as well as postprandial fullness, early satiation, and epigastric pain, were approximately <49% of energy from carbohydrates, >14.7% of energy from proteins, and >37.7% of energy from fats.

### 3.5. Calculated Levels of Macronutrients to Prevent UD Symptoms

Calculated macronutrient intakes for participants with UD to *prevent* symptoms are shown in Table 7. These were >48.2% of energy from carbohydrates, <14.6% of energy from protein, and <38.6% of energy from fat, for postprandial fullness, early satiation, and epigastric pain.

## 4. Discussion

The key findings from this large-scale, cross-sectional study were that (1) participants with UD had higher dietary intakes of fat and lower intakes of carbohydrates, compared with healthy people, and (2) individuals with the highest dietary fat intake were more likely to report postprandial fullness and those with the highest protein intake were more likely to report epigastric pain, compared with those with the lowest intakes. Using the collected data, we also attempted to calculate thresholds for intake levels that would, on the one hand, be able to predict, or, on the other, prevent, UD and found that (3) to *predict* UD in the general population, macronutrient intakes would need to be <49% of energy from carbohydrate and >14.7% and >37.7% of energy from protein and fat, respectively, and (4) to *prevent* symptoms in participants with UD, macronutrient intakes would need to be >48.2% of energy from carbohydrate and <14.6% and <38.6% of energy from protein and fat, respectively. The practical relevance of these cutoffs remains to be determined.

It is now well-recognised that dietary factors play an important role in the pathophysiology of functional dyspepsia, triggering, or exacerbating, symptoms, including postprandial fullness, early satiation, and epigastric pain. Based on frequent reports by patients that certain foods, including fatty foods, wheat- and gluten-containing foods, spicy foods, alcohol, etc., worsen their symptoms [26,27,28,29,30,31,32], common dietary recommendations in clinical practice are to avoid these foods, in an attempt to manage symptoms. Early reports that foods that are rich and high in fat frequently trigger symptoms, sparked interest in the evaluation of the specific role of dietary fat [18,33,34]. Indeed, laboratory-based studies have demonstrated that duodenal infusion of fat, but not glucose, had the capacity to exacerbate fullness and bloating and increase the sensitivity to gastric distension in patients with FD [15]. Moreover, a high-fat meal induced more symptoms, particularly nausea and pain, than an isocaloric high-carbohydrate meal [13], suggesting that symptom induction may indeed be specific to fat.

Surprisingly, few studies have compared the macronutrient composition of the diet between people with FD in free-living populations and healthy people [26,27,29,32], let alone the temporal relationship with symptoms, and their outcomes have not been clear-cut. For example, in one study, fat intake was reported to be higher and carbohydrate intake to be lower in patients than in controls, whereas total energy intake did not differ between patients and controls, although both IBS and FD patients were included in the study [32]. In contrast, two studies reported a reduction in fat intake and an increase in carbohydrate intake in patients with FD, compared with healthy controls [27,29]. In the current study, dietary intake of fat was somewhat higher and carbohydrate intake lower in people with UD, although overall intakes were broadly within recommended dietary intakes [35], except for fat which somewhat exceeded recommendations. This suggests that intakes of specific nutrients do not have to deviate substantially from ‘normal’ intakes to lead to dyspeptic symptoms in people with UD. It is possible that, in some of the studies, patients had adjusted their intakes in an attempt to alleviate symptoms. Nevertheless, we found in our previous prospective study on 20 patients with FD that fullness after meals was related directly to intake of both fat and protein, and inversely to carbohydrates [29]. The data from the current study on a very large sample size of 4763 Iranian adults, which, to our knowledge, is the largest population-based study in the field, seem to confirm our previous findings. Thus, intakes of >35% fat or >20% protein were associated with a non-significantly increased risk of UD, while an intake of >55% carbohydrate was associated with a non-significantly decreased risk of UD. Moreover, individuals with the ‘highest’ fat or protein intakes were more likely to report postprandial fullness or epigastric pain, respectively, compared with those with the ‘lowest’ intakes. Thus, our findings confirm that both fat and protein appear to trigger symptoms, suggesting that carbohydrates may be protective and clearly underlining the importance of the dietary macronutrient composition, both for the induction as well as the prevention or management of dyspeptic symptoms.

The mechanism(s) that may underlie the observed effects are most likely multifactorial. It is known that both fat and protein potently stimulate the gut hormone, cholecystokinin (CCK), while carbohydrates are much less effective in healthy people [36], and plasma CCK responses to a high-fat meal have been found to be greater in patients with FD than in healthy participants [13]. CCK can induce dyspeptic symptoms when administered intravenously in FD patients [37]. Moreover, the CCK receptor antagonist, dexloxiglumide, improved symptoms induced by intraduodenal lipid infusion in FD patients [38]. Thus, it is possible that CCK, at least in part, underlies the induction of symptoms after fat- or protein-containing meals.

Our observation that a high carbohydrate intake may be protective from dyspepsia symptoms is in line with the results of previous reports indicating that acute carbohydrate challenges did not increase symptoms [13,15]. While some studies suggest that certain carbohydrates, particularly fermentable carbohydrates (so-called FODMAPs), may increase symptoms [39], more research is required in this area, although the current data, despite not specifically investigating the contribution of FODMAPs, do not indicate a role.

Given the evidence that higher intakes of specific dietary macronutrients may be associated with dyspeptic symptoms, the question arises whether thresholds could be determined, to either predict or prevent symptoms of dyspepsia. Thus, an additional aim of our study was to attempt to determine the dietary macronutrient composition that may *predict* the occurrence of UD across the study population, while a second question related to a dietary macronutrient composition that might help to *prevent* dyspepsia symptoms in people with UD. Thus, using the dietary data obtained from 4763 participants, we calculated that dietary fat intake should be below 38% of energy to prevent dyspeptic symptoms. This contrasts with previous studies [26,27,29,32], in which patients reported symptoms with lower fat intakes of ~30–34%, although intakes can range from ~15% to 45% [29]. It should be noted that our study was an observational study, and the dietary intakes of participants were evaluated with a self-reported questionnaire and using household measures. Moreover, the severity of dyspeptic symptoms, which was not included in the current analysis, may have been modest in the majority of individuals, given that this was a population-based study. Lifestyle diseases, including obesity, metabolic syndrome, and cardiovascular disease are often associated with dyspeptic/GI symptoms, and we cannot exclude the possibility that symptoms in some of the study participants were due to these diseases. Finally, given that patients with FD or UD may not necessarily have intakes of specific macronutrients that differ from those of healthy individuals (as some previous studies have indicated [32]), but, as discussed, may display a heightened sensitivity to culprit nutrients, with large interindividual differences, it may not be possible to define a specific threshold that would be applicable across a patient cohort. Thus, this approach requires further investigation.

Some limitations of our study should be noted. Due to the cross-sectional design of our study, causality cannot be inferred in our findings. In addition, although we controlled our data for several potential confounders, residual confounding cannot be excluded. Since dyspepsia was not diagnosed based on clinical investigations but determined based on the GI symptoms reported by individuals, as discussed, we cannot exclude that, at least in some individuals, dyspeptic symptoms were related to other causes, such as H pylori and ulcers, etc. We did not quantify the different types of dietary fat or carbohydrate (including fibre) in the dietary analysis or any potential association with dyspeptic symptoms; this warrants evaluation in future studies. Due to the lack of specific Iranian food composition tables, we used the USDA nutrient database to compute daily intakes of nutrients. The study was conducted on staff members of a medical university; therefore, generalisation of our findings to the overall population must be carried out with caution. It is important to bear in mind that the sensitivity to dietary nutrients can vary substantially between individuals; thus, a population-based approach in terms of dietary recommendations may not be suitable. Accordingly, this approach requires validation in well-designed, prospective studies with careful monitoring of macronutrient intakes. Finally, since collecting the data used in the current study approximately 10 years ago, recent evidence suggests modest changes in dietary habits in the Iranian population [40]. While it is important to recognise that changes in dietary habits may potentially affect the outcome of the analyses performed in the current study, such changes are unlikely to impact the validity of our findings on the relationship between dietary nutrient intakes and symptom generation.

In conclusion, our findings demonstrate macronutrient-specific associations with dyspeptic symptoms, particularly postprandial fullness and epigastric pain, highlighting the major roles of fat and protein. Prospective studies designed to carefully manipulate the macronutrient composition of diets are now warranted to investigate the value of dietary changes, including specific macronutrient manipulations, to improve or prevent symptoms in patients with UD.

## Figures and Tables

**Table 1:** General characteristics of the study population.

Variable	With UD	Without UD	*p* Value *
Age (years)	36.1 ± 7.7	36.3 ± 7.8	0.50
Weight (kg)	66.5 ± 13.7	69.0 ± 13.1	<0.001
BMI (kg/m^2^)	24.5 ± 4.3	24.9 ± 3.7	0.02
Female (%)	66.2	56.9	<0.001
Married (%)	82.5	81.6	0.36
University graduate (%)	53.7	63.3	<0.001
High socioeconomic status (%)	23.4	30.9	0.002
Current smoker (%)	16.4	13.4	0.07
Physically active (%)	11.3	13.5	0.17

Data are expressed as means ± SD unless indicated otherwise. * *p* values were obtained using independent Student’s *t*-test or χ2 test, as appropriate. UD, uninvestigated dyspepsia.

**Table 2:** Dietary intakes of the study population.

Variable	With UD	Without UD	*p* Value *
Energy (kcal)	2323 ± 822	2388 ± 826	0.10
Carbohydrate (% energy)	48.2 ± 8.1	49.2 ± 7.8	0.01
Protein (% energy)	14.8 ± 2.5	14.8 ± 2.3	0.76
Fat (% energy)	38.3 ± 6.5	37.4 ± 6.5	0.007

Data are expressed as mean ± SD. * *p* values were obtained using independent Student’s *t*-test. UD, uninvestigated dyspepsia.

**Table 3:** Prevalence of uninvestigated dyspepsia and its symptoms in relation to macronutrient intakes.

	Carbohydrate (% Energy)			*p* Value *	Protein (% Energy)			*p* Value *	Fat (% Energy)			*p* Value *
	≤50	50–55	>55		≤15	15–20	>20		≤25	25–35	>35	
Participants with UD (%)	15.3	13.8	13.3	0.33	14.7	13.8	22	0.12	12.3	13.1	15.3	0.20
Postprandial fullness (%)	38	38	35.5	0.45	38.5	35.7	43.9	0.12	28.3	35.8	38.6	0.04
Early satiation (%)	28.2	27.3	24.8	0.20	27.3	26.9	32.9	0.48	24.5	25.4	28.2	0.20
Epigastric pain (%)	39.1	39	37.8	0.83	39.7	37	50	0.02	41.5	37.2	39.4	0.43

* *p* values were obtained using χ^2^ test. UD, uninvestigated dyspepsia.

**Table 4:** Odds ratios and 95% confidence intervals in participants with uninvestigated dyspepsia and its symptoms according to categories of macronutrient intakes.

	Carbohydrate (% Energy)			*p*-Trend	Protein (% Energy)			*p*-Trend	Fat (% Energy)			*p*-Trend
	≤50	50–55	>55		≤15	15–20	>20		≤25	25–35	>35%	
	OR	OR	OR		OR	OR	OR		OR	OR	OR	
		(95% CI)	(95% CI)			(95% CI)	(95% CI)			(95% CI)	(95% CI)	
**Uninvestigated dyspepsia**												
Crude	1	0.88	0.84	0.15	1	0.93	1.63	0.81	1	1.07	1.28	0.08
		(0.69, 1.12)	(0.66, 1.08)			(0.76, 1.13)	(0.95, 2.70)			(0.58, 1.97)	(0.71, 2.33)	
MV *	1	1	0.86	0.37	1	1.02	1.35	0.61	1	1.05	1.19	0.3
		(0.75, 1.34)	(0.64, 1.16)			(0.80, 1.29)	(0.65, 2.77)			(0.50. 2.46)	(0.58, 2.46)	
**Postprandial fullness**												
Crude	1	1	0.89	0.27	1	0.88	1.25	0.35	1	1.14	1.5	0.02
		(0.84, 1.18)	(0.75, 1.07)			(0.77, 1.02)	(0.80, 1.95)			(0.91, 2.20)	(1.03, 2.44)	
MV *	1	0.97	0.96	0.69	1	0.88	0.98	0.24	1	1.37	1.47	0.19
		(0.79, 1.20)	(0.77, 1.18)			(0.74, 1.05)	(0.55, 1.73)			(0.79, 2.37)	(0.86, 2.5)	
**Early satiation**												
Crude	1	0.95	0.83	0.08	1	0.97	1.3	0.81	1	1.05	1.2	0.83
		(0.79, 1.15)	(0.69, 1.01)			(0.83, 1.14)	(0.81, 2.09)			(0.66, 1.67)	(0.77, 1.90)	
MV *	1	0.9	0.84	0.14	1	1.11	1.35	0.17	1	0.79	0.95	0.18
		(0.72, 1.14)	(0.66, 1.06)			(0.92, 1.35)	(0.72, 2.52)			(0.45, 1.38)	(0.55, 1.64)	
**Epigastric pain**												
Crude	1	0.99	0.95	0.59	1	0.89	1.51	0.6	1	0.83	0.91	0.51
		(0.84, 1.18)	(0.79, 1.13)			(0.77, 1.02)	(0.97, 2.36)			(0.55, 1.25)	(0.61, 1.35)	
MV *	1	0.94	0.96	0.98	1	0.93	1.48	0.92	1	0.9	0.93	0.92
		(0.76, 1.16)	(0.78, 1.19)			(0.78, 1.10)	(0.84, 2.58)			(0.54, 1.49)	(0.57, 1.52)	

MV, multivariable adjustment; OR, odds ratio; CI confidence interval. * Adjustment for age, sex, energy intake, physical activity, marriage status, education, socioeconomic status, smoking, and BMI.

**Table 5:** Area under receiver operating characteristic curve for uninvestigated dyspepsia and its symptoms by individual macronutrients.

	Carbohydrate	Protein	Fat
	AUC (95% CI)	AUC (95% CI)	AUC (95% CI)
Uninvestigated dyspepsia	0.47 (0.44, 0.49)	0.48 (0.48, 0.51)	0.53 (0.50, 0.56)
Postprandial fullness	0.48 (0.46, 0.50)	0.48 (0.46, 0.50)	0.52 (0.50, 0.54)
Early satiation	0.47 (0.45, 0.49)	0.48 (0.46, 0.50)	0.53 (0.51, 0.55)
Epigastric pain	0.49 (0.47, 0.51)	0.47 (0.45, 0.49)	0.50 (0.48, 0.52)

AUC, area under the ROC curve; CI confidence interval.

**Table 6:** Calculated macronutrient intakes to predict uninvestigated dyspepsia and its symptoms in generally healthy adults.

	Carbohydrate (% of Energy)	Protein (% of Energy)	Fat (% of Energy)
Uninvestigated dyspepsia	48.8	14.7	38.0
Postprandial fullness	49.1	14.7	37.7
Early satiation	48.8	14.7	37.8
Epigastric pain	49.0	14.7	37.7

**Table 7:** Calculated macronutrient intakes to prevent symptoms of uninvestigated dyspepsia in individuals with the condition.

	Carbohydrate (% of Energy)	Protein (% of Energy)	Fat (% of Energy)
Postprandial fullness	48.2	14.6	38.6
Early satiation	48.6	14.6	38.5
Epigastric pain	48.4	14.5	38.3

## Data Availability

The data presented in this study are available on request from the corresponding author. The data are not publicly available due to privacy reasons.
